# Experimental Evaluation of Kidney Regeneration by Organ Scaffold Recellularization

**DOI:** 10.1038/srep43502

**Published:** 2017-03-07

**Authors:** Andrea Remuzzi, Marina Figliuzzi, Barbara Bonandrini, Sara Silvani, Nadia Azzollini, Roberta Nossa, Ariela Benigni, Giuseppe Remuzzi

**Affiliations:** 1 IRCCS - Istituto di Ricerche Farmacologiche Mario Negri, Centro Anna Maria Astori Via Stezzano 87 - 24126 Bergamo, Italy; 2 Department of Management, Information and Production Engineering, University of Bergamo, Viale Marconi 5 - 24044 Dalmine Bergamo, Italy; 3 Unit of Nephrology and Dialysis, Azienda Ospedaliera Papa Giovanni XXIII Piazza OMS 1 – 24127 Bergamo, Italy; 4 Department of Biomedical and Clinical Sciences, University of Milano, Via Festa del Perdono 7 -20122 Milano, Italy

## Abstract

The rising number of patients needing renal replacement therapy, alongside the significant clinical and economic limitations of current therapies, creates an imperative need for new strategies to treat kidney diseases. Kidney bioengineering through the production of acellular scaffolds and recellularization with stem cells is one potential strategy. While protocols for obtaining organ scaffolds have been developed successfully, scaffold recellularization is more challenging. We evaluated the potential of *in vivo* and *in vitro* kidney scaffold recellularization procedures. Our results show that acellular scaffolds implanted in rats cannot be repopulated with host cells, and *in vitro* recellularization is necessary. However, we obtained very limited and inconsistent cell seeding when using different infusion protocols, regardless of injection site. We also obtained experimental and theoretical data indicating that uniform cell delivery into the kidney scaffolds cannot be obtained using these infusion protocols, due to the permeability of the extracellular matrix of the scaffold. Our results highlight the major physical barriers that limit *in vitro* recellularization of acellular kidney scaffolds and the obstacles that must be investigated to effectively advance this strategy for regenerative medicine.

Rapidly rising global rates of chronic diseases, such as diabetes and hypertension, portend a consequent rise in end-stage renal disease (ESRD)^1^. Renal replacement therapy (RRT), through either dialysis or renal transplantation, is a lifesaving but very costly treatment for people with ESRD^1^. The global prevalence of maintenance dialysis has increased 1.7 times from 165 pmp patients in 1990 to 284 pmp in 2010^1^. Moreover, it has been estimated that the expected number of people receiving RRT (dialysis or transplantation) will more than double from 2.6 billion people worldwide in 2010 to 5,4 billion in 2030^2^. Notably, between 2,3 and 7,1 billion people who could have been kept alive with RRT in 2010 died prematurely because they did not have access to treatment^2^. Most of these deaths occurred in low-income and middle-income countries in Asia, Africa, and Latin America, where RRT remains unaffordable for the majority of affected people and causes severe financial hardship for those who have access to it^3^.

For the foreseeable future, in view of the expected increase in the number of patients who will need treatment, dialysis provision will be a substantial financial burden for even the most affluent countries, given that dialysis techniques currently cost tens of thousands of US dollars per patient per year. Renal transplantation is recognized as the best available treatment for ESRD in terms of cost, quality of life, and survival^4^,^5^. However, shortages of deceased donor organs for transplantation limit this treatment option worldwide^5^.

To overcome the limits of current RRT, numerous investigators have suggested that tissue engineering may be a promising approach for regenerating damaged kidneys *in vitro*^6^,^7^. Theoretically, producing a decellularized kidney scaffold and then seeding it with stem cells to repopulate the kidney’s extracellular matrix (ECM)^8^,^9^ could be a promising technique. Initial, promising results, especially in kidney tissue decellularization, have generated great expectations not only in researchers^10^,^11^ but also in the general public^12^. However, after obtaining promising results with kidney decellularization, scientists have started to realize that effectively advancing the recellularization of natural scaffolds is more complex than was previously hoped, and that it necessitates the development of novel techniques to obtain uniform cell seeding and growth within acellular tissues^13^,^14^,^15^ and this is particularly challenging for the peculiar 3D structure of the renal vasculature and tubules.

As expected, the structural complexity of the scaffold, and the variety of cells in the kidney, make the repopulation of kidney scaffolds a rather difficult task. Despite these difficulties, there are several recent reports in the literature of experimental methods being used to repopulate acellular kidney scaffolds. Cell seeding within these scaffolds has been performed by perfusing cell suspensions through either the renal artery or the ureter^8^,^16^,^17^. Ross *et al*. were the first to report the recellularization of rat kidney scaffolds with murine embryonic stem (mES) cells, manually perfused through the renal artery and the ureter^8^. In similar studies, we reported that acellular kidney scaffolds were recellularized using mES cells via infusion through the renal artery^17^. Seeded cells were distributed uniformly throughout the vascular network and mostly in glomerular capillaries, but they only occasionally reached peritubular capillaries with, as expected, no cells in the tubular lumen. To obtain cell engraftment in the renal tubules and in the collecting system, Song *et al*.^16^ infused neonatal rat kidney cells through the ureter while a negative pressure gradient was applied to the organ chamber. Using this technique, some cells were distributed throughout the tubular compartment, up to the glomerular tuft. In order to improve endothelial cell seeding throughout the kidney vasculature, Ko *et al*.^18^ pretreated the ECM with a CD31 antibody to favor the efficient adhesion of endothelial cells to the vessel wall. More recently, Caralt *et al*.^9^ infused a higher number of human renal cortical tubular epithelial cells in rat kidney scaffolds at a higher pressure than is usually adopted, and obtained up to 50% coverage in some parts of the renal parenchyma 24 hours after seeding.

Though these preliminary attempts were somewhat encouraging^11^,^19^,^20^,^21^,^22^,^23^, a more careful evaluation of the studies available indicates that there is still a long way to go before *in vitro* organ regeneration can be achieved. The aim of our study, then, was to assess the actual potential of kidney scaffold recellularization procedures for future clinical applications, and to identify the obstacles that we need to overcome. We initially investigated whether the implantation of acellular scaffolds in the rat could start the process of recellularization. Since these preliminary experiments showed that implanted scaffolds do not repopulate with cells from the recipient, even three weeks after implantation, we then evaluated strategies for *in vitro* cell seeding with different experimental protocols, as previously suggested in the literature^9^,^16^, to obtain the cellularization of the different structural compartments of kidney scaffolds. The aim was to establish the real potential of kidney scaffold recellularization techniques, using embryonic stem cells due to their potential to proliferate, as well as to differentiate when they are in intimate contact with ECM proteins of basement membranes along the nephron. We also investigated the physical barriers that recellularization protocols come up against when attempting to obtain uniform and extended cell seeding in acellular rat kidney scaffolds.

## Results

### Orthotopic transplantation of acellular scaffolds

**Figure 1:**
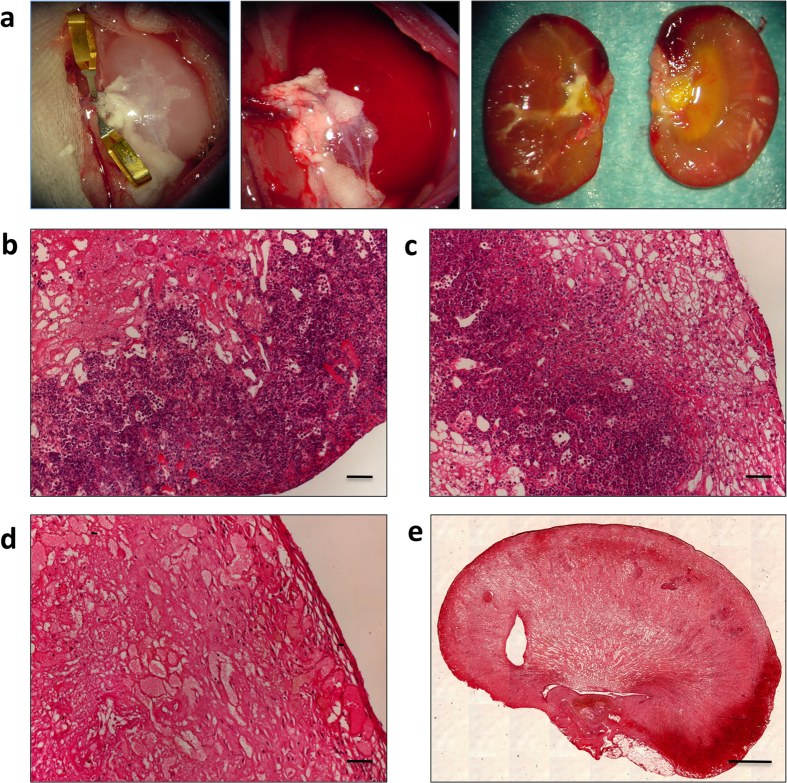
Explanted renal scaffolds. (**a**) Photograph of the transplanted decellularized kidney before (left) and after (middle) unclamping of left renal artery and vein showing homogeneous perfusion of the graft without signs of bleeding. (**a**, right) Photograph of longitudinal sections of transplanted scaffold after three weeks within Lewis recipient rats. (**b**–**e**) Hematoxylin and eosin staining of the explanted scaffolds demonstrates the presence of intense inflammatory cell infiltration, especially in the renal pericapsular areas and the remaining structures are filled with clotted material (**b** and **c**, 3 and 7 days, respectively). At 21 days the acellular scaffolds show less cell infiltration and some ECM degradation (**d** and **e**). (**b**,**c**,**d**) Scale bar, 50 μm. (**e**) Scale bar, 1000 μm.

Rat kidneys were decellularized by perfusion through the renal artery using the protocol we previously reported^17^. Complete cell removal was obtained within 6 hrs through SDS infusion of the kidneys under perfusion pressure (on average 109 ± 22 mmHg), close to the physiological value in the rat (110–115 mmHg)^24^. Using optical microscopy we observed that the decellularization protocol yielded intact scaffold microarchitecture for the glomeruli and tubules, and that the integrity of blood vessels had been maintained (data not shown). To investigate whether, after initial blood clotting in the denuded vascular wall, host cells could possibly repopulate the kidney scaffold, we implanted the scaffolds into Lewis recipients in an orthotopic position, partially preventing thrombus formation through pharmacological treatment. The left renal artery and left renal vein of the recipient were successfully anastomosed to the decellularized kidney’s renal artery and vein, respectively (as shown in Fig. 1). After the vascular microclamps were removed, blood flowed uniformly throughout the entire implanted scaffolds (Fig. 1a). The scaffold implantation was tolerated well in animals and no adverse reactions were noted. Recipient animals were sacrificed at 3, 7 and 21 days after implantation. At 3 and 7 days explanted kidneys showed significant clotting that appeared to have been partially reabsorbed at 21 days. Macroscopic examination of the longitudinally sectioned grafts revealed full preservation of the gross anatomy, and both the cortex and medulla were well demarcated (Fig. 1a, right). Hematoxylin and eosin staining showed massive infiltration of cells, predominantly in the outer layer of the scaffold (Fig. 1b). The cell infiltration was still present 7 days after implantation, with obstruction of the vasculature tree by entrapped red blood cells (Fig. 1c). At 21 days after implantation, inflammatory cells decreased notably, without repopulation of the ECM with cells from the host in the original kidney scaffold, and uniform reabsorption of ECM and shrinkage of the entire scaffold was noted (Fig. 1d and e). The dynamic changes of implanted acellular scaffolds indicated that cellular repopulation does not occur in the scaffold vasculature and parenchyma over time, while the scaffold ECM matrix starts to be degraded and reabsorbed.

### Cell seeding with mES cells

**Figure 2:**
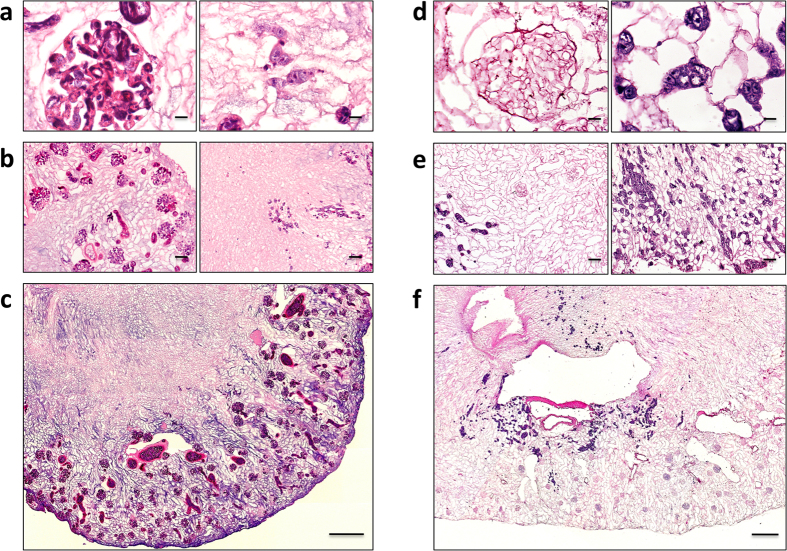
Repopulation of kidney scaffolds with mES cells via RA and RV for 24 hours. Hematoxylin and eosin staining of representative kidney scaffold seeded with mES cells via the RA (**a**–**c**) shows that cells are located into glomerular (**a** and **b**, left) and occasionally in peritubular capillaries (**a** and **b**, right). (**a**) Scale bar, 10 μm. (**b**) Scale bar, 50 μm. (**c**) Mosaic view of a transversal cross section of kidney recellularized by RA demonstrating a homogeneous repopulation of glomeruli and vascular structures in the cortical region. Scale bar, 250 μm. (**d**–**f**) Hematoxylin and eosin staining of kidney scaffold seeded with mES cells via RV shows that glomeruli are devoid of cells (**d** and **e**, left). mES cells are preferentially located in peritubular capillaries in the medulla (**d** and **e**, right). (**d**) Scale bar, 10 μm. (**e**) Scale bar, 50 μm. (**f**) Mosaic view of a transversal cross-section of kidney recellularized via RV with cells only focally an sparsely located within the scaffold. Scale bar, 250 μm.

**Figure 3:**
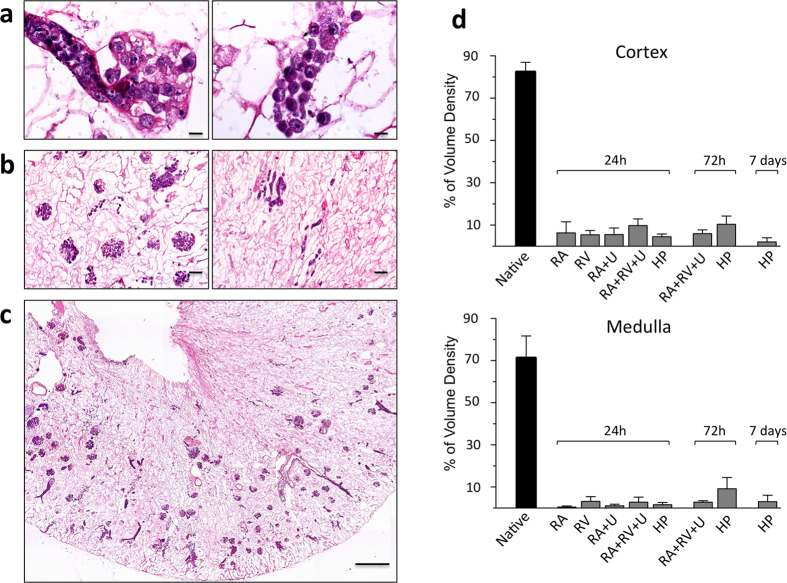
Recellularization of kidney scaffolds with mES via RA and U for 24 hours and quantification of seeded cells in kidney scaffolds. (**a**–**c**) Hematoxylin and eosin staining of kidney scaffold seeded with mES cells via RA and U shows that cells are located in glomerular and vascular structures in the cortical region (**a** and **b**, left), while cells infused through the collecting system partially reached tubular compartments (**a** and **b**, right). (**a**) Scale bar, 10 μm. (**b**) Scale bar, 50 μm. (**c**) Mosaic view of a transversal cross section of recellularized kidney demonstrating that most cells were confined to the glomerular capillary level and very few cells reached the tubular compartment. Scale bar, 500 μm. (**d**) Volume density of seeded cells in cortical (upper) and medullar (lower) regions after infusion of mES cells with different experimental protocols (n = 5 for RA; n = 3 for RV, RA + U, RA + RV + U and HP) and different time points compared to native untreated kidneys (n = 3).

After concluding that *in vivo* kidney tissue repopulation through the implantation of the acellular scaffold was not feasible, we investigated different experimental strategies for the *in vitro* cell repopulation of acellular kidney scaffolds. Initially, we evaluated the distribution of mES cells infused into the renal artery (RA) and maintained in culture for 24 hours. As shown in Fig. 2(a–c), seeded cells homed primarily to the cortical region of the kidney, where they were distributed uniformly in the arterial structures and in glomerular capillaries. Cells were mostly trapped in the glomerular capillaries, with some arteriolar localization (Fig. 2a and b, left). We only occasionally observed cells in the peritubular capillaries (Fig. 2a and b, right). Almost no cells arrived in the medullar compartment (Fig. 2c) and, more importantly, the amount of scaffold volume occupied by the cells was very low (Fig. 2c). As shown in Fig. 3d, we used point counting morphometry to estimate that the percentage of volume occupied by cells (volume density) averaged only 6.31 ± 5.27% in the kidney cortex and 0.48 ± 0.53% in the medullar area. This is far lower than the volume occupied by cells in native kidneys, which we estimated to average 81.8 ± 3.7% and 73.0 ± 8.6% in the cortex and medulla. We also performed additional experiments (data not shown) with lower cell numbers, and a lower cell suspension volume and infusion rate. The degree of recellularization and cell localization were always worse than reported above.

To obtain cell seeding in the venous compartment, we infused mES cells through the renal vein (RV) and, as expected, observed a different trend (as shown in Fig. 2d–f). At 24 hours post-seeding, cells were present predominantly in the medulla, and located in peritubular capillaries (Fig. 2d and e, right), while no cells were present in glomeruli (Fig. 2d and e, left). That cells were observed almost exclusively in the peritubular capillary is shown by the 3D view of serial H&E sections (3 μm) reported in the additional material (Figure A2) in which empty tubular lumens are visible and cells are interposed among them. However, as shown by a representative experiment in Fig. 2f, the distribution of cells was not uniform, with focal areas occupied by cells, but in the majority of the scaffold cells were absent. Using this perfusion protocol, the volume density of cells seeded in the cortex averaged 5.32 ± 1.92%, and 2.56 ± 2.15% in the medulla (Fig. 3d).

In an attempt to deliver the cells into the renal collecting system too, we perfused mES cells via the RA and the ureter (U). As shown in Fig. 3 (a–c), cell seeding through the arterial vasculature enabled cell engraftment in the renal cortex, as expected, while cells infused through the U traveled through the collecting ducts and arrived in the tubular structures. However, the number of infused cells observed in the tubular compartment was very low, and they were distributed sparsely across the scaffold. The volume density of seeded cells with this infusion protocol was 4.70 ± 2.81% and 0.92 ± 0.75% in the cortex and medulla, respectively (Fig. 3d).

**Figure 4:**
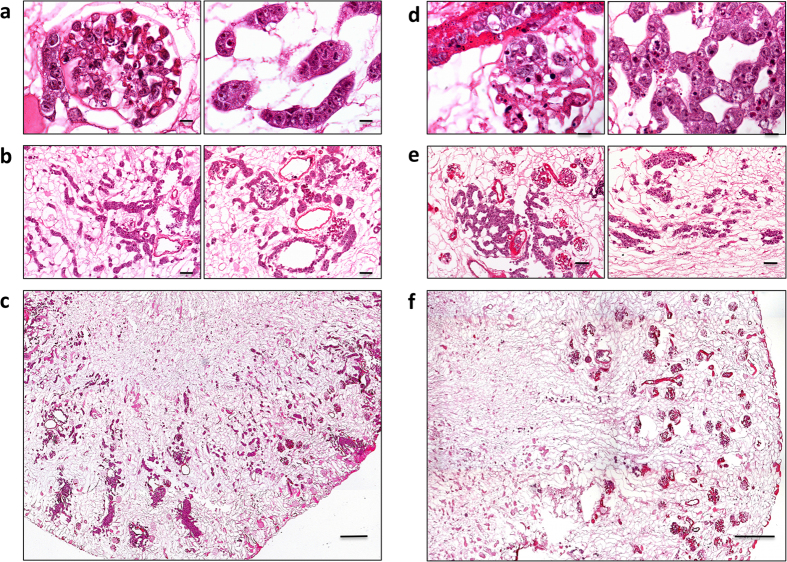
Recellularization of kidney scaffolds with mES via RA, RV and U for 24 hours and 72 hours. (**a**–**c**) Hematoxylin and eosin staining of kidney scaffold seeded with mES cells via the RA, RV, and U shows cells located in glomerular, vascular and tubular structures, at 24 hours. (**c**) Mosaic view of a transversal cross section of recellularized kidney demonstrating that most cells were confined to the cortical region and very few cells reached the medullar region. (**d**–**f**) Hematoxylin and eosin staining of kidney scaffold seeded with mES cells via RA, RV and U and continuously perfused with recirculating culture medium for 72 hours. Cells are located in glomerular, vascular structures with a pattern similar to 24 hours. (**f**) Mosaic view of a transversal cross section of recellularized kidney. (**a** and **d**) Scale bar, 10 μm. (**b** and **d**) Scale bar, 50 μm. (**c** and **f**) Scale bar, 250 μm.

To increase cell seeding efficiency, we also adopted a combined approach, recellularizing renal scaffolds by infusing mES cells through the RA, the RV and the U. As shown in Fig. 4(a–c), the results confirmed that the combined infusion (RA + RV + U) strategy resulted in improved cellular delivery, with more homogeneous cell distribution compared to the single or double infusions at 24 hours. Volume density was 8.90 ± 2.50% and 2.21 ± 1.80% in the cortex and medulla, respectively (Fig. 3d).

To investigate whether seeded cells could proliferate and occupy a larger volume fraction of the scaffold we compared cellular volume density at different perfusion times after cell seeding of the scaffolds by arterial, venous and ureter infusion. However, as shown in Fig. 4(d–f), the volume occupied by cells at 72 hours decreased, rather than increasing over time. As shown in Fig. 3d, actual volume density was 5.19 ± 1.77% and 2.23 ± 1.22% in the cortex and medulla, respectively. These data are in line with the results from Ki-67 staining (see Table 1), that show that mean percentage of proliferating cells was significantly lower at 72 hours as compared to 24 hours (37.7 ± 4.9% vs. 57.5 ± 3.1%, respectively, p < 0.01).

**Figure 5:**
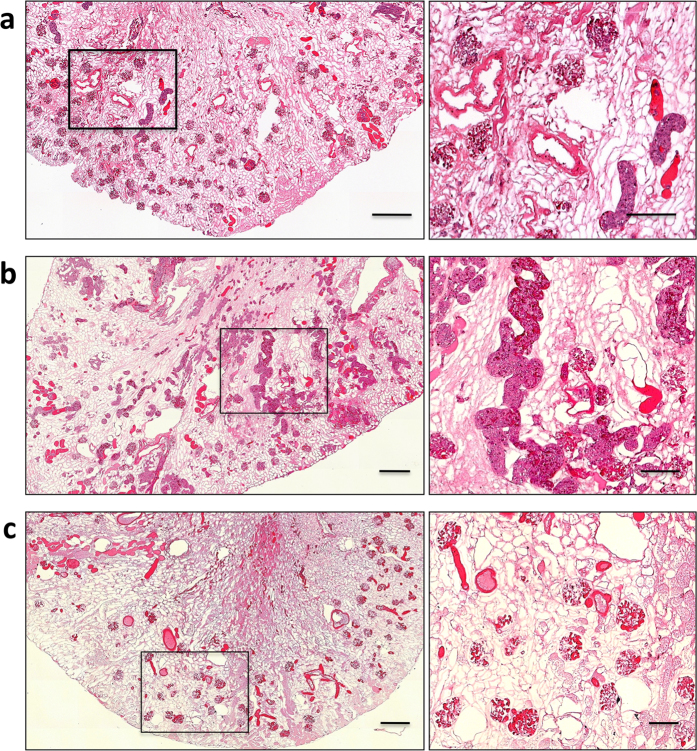
Recellularization of kidney scaffolds with mES via RA at high pressure (HP). Mosaic view of transversal cross sections of hematoxylin and eosin stained recellularized kidneys 24 hours, 72 hours and 7 days after seeding. (**a**) The cells have major distribution within the tubules at 24 hours post-seeding despite being injected through the arterial vasculature. (**b**) The repopulation of scaffolds was increased 72 hours post-seeding, (**c**) while there was a decrease in cell numbers after 7 days of perfusion. (**a**,**b**,**c**, left) Scale bar, 250 μm. (**a**,**b**,**c**, right) Scale bar, 125 μm.

We finally considered an alternative approach to recellularizing kidney scaffolds by infusing mES cells via the renal artery, as in a recently published paper^9^, infusing a higher number of cells at a higher pressure and flow rate. As shown in representative Fig. 5, at 24 hours post-seeding, cell distribution within the tubules was slightly better, likely due to the translocation of mES cells from vessel matrices into the peritubular spaces (Fig. 5a). Cell volume density averaged 4.92 ± 2.05% and 1.88 ± 1.18% in the cortex and medulla, respectively (Fig. 3d). However, since single cell suspensions were infused, the morphology of observed cell clusters indicates that the high-pressure perfusion may have ruptured the original blood vessel membrane focally, and cells started to accumulate in clusters within tubular basement membranes (Fig. 5b). Actually, cell volume density increased time-dependently between 24 and 72 hours of perfusion, with cell volume densities averaging 9.34 ± 4.10% and 8.75 ± 4.20% in the cortex and medulla, respectively. However, a decrease in seeded cells was observed after 7 days of medium perfusion (as shown in Fig. 5c) with a cell volume density of 4.16 ± 3.30% and 4.69 ± 3.40% in the cortex and medulla, respectively (see Fig. 3d). These results suggest that cells were viable and underwent proliferation within the scaffold both at 24 and 72 hours (as shown in Table 1). The mean percentage of proliferating cells averaged 50.9 ± 11.2%, and 51.8 ± 2.1% at 24 and 72 hours, respectively. However, later in time the mean percentage of proliferating cells was significantly lower and averaged only 23.7 ± 13.1% at 7 days after cell seeding (p < 0.05 vs. 24 hrs, p < 0.01 vs. 72 hrs).

### Cell and medium distribution during cell seeding

**Figure 6:**
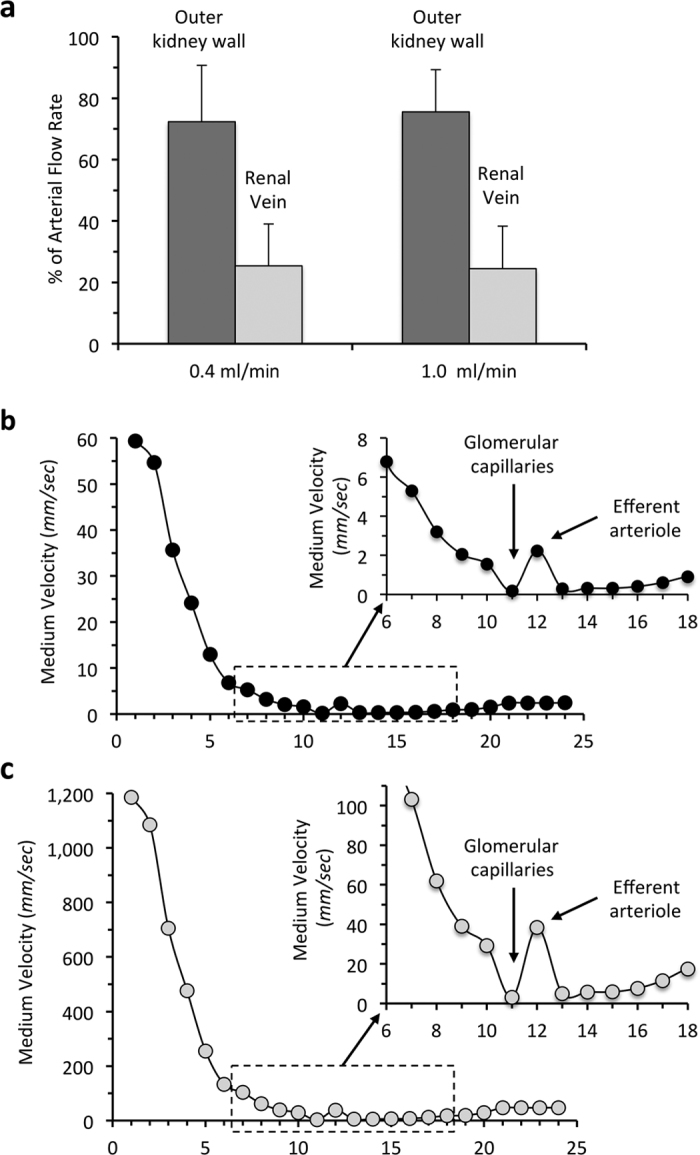
Fluid flow and velocity distribution in acellular kidney scaffolds. (**a**) Measurements of perfusion medium exiting from the renal vein as compared to fluid exiting from the ureter and kidney capsule during arterial infusion. (**b**) Calculated fluid velocity of cell perfusion medium along the entire scaffold, from renal artery to the renal vein for infusion of cell medium at 1.0 ml/min. (**c**) Calculated fluid velocity in the scaffold circulation for infusion of cell medium at 20 ml/min.

The perfusion of cell suspensions through the renal artery was performed with the aim of obtaining uniform cell seeding in the renal scaffolds. This is based on the assumption that the fluid perfused is distributed uniformly in the arterial circulation, microcirculation, and up to the venous outflow. However, the wall of the vasculature and that of the tubular membrane of the scaffold are expected to be permeable to water, and to allow some degree of filtered fluid to leave the blood vessel lumen and even to escape from the tubular compartment. We then measured the amount of cell suspension medium that exited through the renal vein when the flow rate we adopted for cell seeding (1.0 ml/min) and *in vitro* culture (0.4 ml/min) was infused into the artery, and then measured that collected from the outer surface of the kidney scaffold. As reported in Fig. 6a, we measured that on average only 25.4 ± 13.6% (n = 7) of the arterial flow rate of 0.4 ml/min exits from the vein, while the remaining fluid (72.4 ± 18.3%) permeates the scaffold ECM and exits from the outer wall (the ureter and eventually the renal capsule). Similar results have been obtained with a flow rate of 1.0 ml/min (n = 7) through the renal artery (only 24.5 ± 13.8% exits from the vein and 75.5 ± 13.8% exits from the ureter and the outer surface). Thus, a large fraction of the flow rate is expected to leave the blood vessels, and as a consequence to reduce the flow rate of the cell suspension in the vascular compartment.

### Theoretical analysis of medium flow velocity during cell seeding

We then used a theoretical model to calculate the effective velocity of the cell suspension media in the different segments of the vasculature of the kidney scaffold during the cell seeding procedure. We adopted the theoretical model described in the Appendix (see Supplementary Material). The model was validated on the basis of the good agreement between calculated values of pressure drop across the entire kidney and our experimental data, as well as with data reported previously^25^ (see Appendix). We then considered vessels, in the model, to be permeable (leaking wall) according to the analytical solution published previously^26^. We initially simulated the average perfusion of the scaffold, assuming an arterial flow rate of 1.0 ml/min, and we changed the perfusion pressure and hydraulic permeability coefficient (*k*) iteratively, which allowed us to fit measured mean value of flow rate into the renal vein and mean total pressure drop. Using the estimated value of *k*, we then calculated the flow distribution for arterial flow rates of 1.0 and 20 ml/min, respectively. From both arterial flow rates we calculated, for each hierarchical vessel level, local flow rates and average cell medium velocity. For the arterial flow rate of 1.0 ml/min, the values are reported in Fig. 6b for the entire arterial and venous vasculature. Due to extensive branching of the arterial tree, and the leakage of fluid due to hydraulic permeability of the vessel wall, after the initial arterial segments the velocity rapidly decreases along the entire arterial tree. In afferent arterioles and in glomerular capillaries the calculated medium velocity reaches a minimum of about 180 μm/sec. Then, as expected, in efferent arterioles the calculated fluid velocity increases due to fluid flow from the glomerular capillary bed joining in a single vessel. In peritubular capillaries the estimated mean velocity of the medium is reduced once again to around 300 μm/sec. These low fluid velocities suggest that during cell infusion into the renal artery the cell suspension must move very slowly along the scaffold microcirculation and the seeding might even stop in glomerular capillaries. These data are consistent with the accumulation of cells observed almost exclusively at the glomerular capillary level, with very few cells present in the tubular microcirculation, at the cortical and medullary level, and along the venous side.

We also calculated fluid flow velocity for the infusion rate of 20 ml/min, which allowed infused cells to arrive in focal areas after the glomerular capillaries, and the results are reported in Fig. 6c. As expected, we calculated higher values along the scaffold circulation for this high flow rate. The average velocity in the glomerular capillaries was calculated to be about 3.0 mm/sec under this perfusion flow rate. This residual velocity might suggest a more favorable flow transport of the cell suspension. However, the experimental morphological results indicate the presence of focal cell clusters in the tubular space. Since single cells in suspension were infused in the renal artery in these experiments, it is likely that, for the perfusion pressure induced by the elevated flow rate, individual cells left the vasculature and re-aggregated in the tubular and peritubular space to form the observed cell clusters, due in particular to the low fluid velocity and pressure present outside that vascular space.

## Discussion

In the last five years there have been a growing number of investigations into the difficult task of *in vitro* organ regeneration^27^,^28^. After the successful development of tissue engineering applications, from the laboratory to the clinic, it has been suggested that a similar approach could have great potential to create functional organs in the laboratory^19^,^29^. The hypothesis is that recellularizing human organ scaffolds with stem cells could open the way for new strategies to overcome organ donation shortages, as well as immune rejection after organ transplantation. This attractive hypothesis has been strengthened by the possibility of reprogramming adult differentiated cells into pluripotent stem cells, with the obvious advantage of avoiding immune rejection^30^.

Setting up fast and efficient procedures for organ decellularization has turned out to be successful^31^,^32^,^33^,^34^. These laboratory procedures allow us to maintain vascular structure and ECM composition^17^, which is believed to be important for inducing and maintaining cell differentiation. There is also evidence that these whole organ scaffolds can successfully be implanted and connected to the vasculature^18^,^35^,^36^. However, as expected, in these studies massive thrombosis developed soon after implantation. We then reasoned that a similar approach with a tighter control of blood coagulation could be worth investigating. To this end we implanted acellular kidney scaffolds in the rat and followed them for up to three weeks. Our data show that, even when we prevent massive coagulation, inflammatory cells that progressively destroy the ECM characterize cell infiltration in the scaffold. These pro-inflammatory cells may also be attracted by the implanted ischemic tissue. Infiltrating cells do not start to recapitulate vascular or tubular structures, but appear to induce the reabsorption of ECM material and, by the end of our observation period, most of the volume of the acellular scaffold had been reabsorbed. ECM reabsorption may also derive from proteolytic activity. Thus, the *in vivo* regeneration of kidney tissue by using denuded ECM is not a feasible strategy, and acellular kidney scaffolds must be recellularized before organ implantation.

Effective cell seeding of entire organ scaffolds is actually a major task. The first attempt in this direction^8^ with the recellularization of rat kidney scaffolds using mES infused into the renal artery showed cell attachment to the scaffold and proliferation that was, however, rather low, and predominantly observed at the glomerular capillary level. The infusion protocol reported more recently by Song *et al*.^16^, infusing endothelial cells and fetal kidney cells through the renal artery and the ureter, respectively, maintaining negative pressure outside the kidney scaffold during cell infusion, allowed cell seeding into the tubular space too. However, a limited amount of cells were observed at the histological level in this study. The authors reported that this rudimentary kidney also produced urine through the ureter, and that a small amount of creatinine clearance was measured. This was interpreted as fractional solute excretion and tubular absorption by the regenerated kidney^16^. However, it is more likely that passive transport of creatinine, due to the permeable ECM of blood capillaries and the tubular membrane, was the reason for the observed excretory function. Despite the experimental problems encountered in these preliminary investigations, high expectations have been created in the literature^19^,^22^,^37^,^38^ suggesting that positive results from ongoing studies will soon make it possible to obtain kidneys regenerated *in vitro* for human transplantation.

In addition to the problem of recellularization, there are other important^39^,^40^ hurdles in the field related to the source of renal cells and the need to obtain, inside the kidney, differentiation of infused cells into numerous phenotypes, as found in the native organ. The general concept is that, using embryonic stem cells, differentiation can be induced through the contact of seeded cells with ECM proteins along the nephron, as well as the 3D structure of the ECM membranes. We recently addressed this issue by recellularizing acellular rat kidney scaffolds with mES cells^17^. We demonstrated that these cells potentially lose the markers of stemness they had in 2D cultures once they are in contact with the acellular matrix. We also demonstrated that mES cells start to express endothelial markers. Thus, theoretically, it is possible to assume that mES cells will undergo differentiation when they remain in contact with the kidney ECM and its 3D structure. In our present investigation we focused on the outcome of renal scaffold recellularization, as this is the main limiting factor at the moment.

Our data clearly demonstrated that the mES we infused in the renal artery arrived in the glomerular capillaries uniformly but, despite the cell suspension medium being infused continuously, almost all of the cells occupy the capillary lumen and do not move further into the peritubular capillary bed and venous circulation. In an attempt to recellularize the venous compartment, we evaluated the effect of mES cell infusion into the renal vein. Using this protocol we observed that some cells arrive in the peritubular capillary bed, at the cortical and medullary level, but only a small proportion of the volume of the scaffolds was seeded by these cells, and this happened only in focal areas. In addition, cells did not reach the glomerular capillary in the retrograde direction, allowing only partial recellularization.

In line with previous protocols^16^, we also evaluated the efficiency of the technique of infusing cells into the ureter, with vacuum applied outside the kidney scaffold. This procedure allowed us to recellularize the tubular compartment, but only for a small volume fraction. In addition, we also evaluated the degree of recellularization obtained when all three infusion routes were used (the artery, the vein and the ureter). However, even under these conditions, cells were confined to focal areas and not uniformly distributed in the cortex and in the medullary volume of the kidney scaffolds.

In an attempt to increase the degree of recellularization, Caralt *et al*.^9^ recently designed a specialized bioreactor to infuse cells in a rat kidney scaffold through the renal artery, perfusing the cell suspension at high pressure. The authors seeded 40 million cells and were able to recellularize about 50% of the renal volume, at 24 hours after cell seeding. However, morphological images from this report showed that cells infused under high pressure translocated out of the microcirculation and extravasated into peritubular structures, likely as a result of capillary and tubular wall rupture. Despite cell extravasation and aggregation, a time-dependent increase in metabolic activity was observed, suggesting persistent cell viability and proliferation within the scaffolds. Based on these results we also evaluated the effect of the high-pressure perfusion protocol during cell seeding. Our data confirmed that this procedure makes it possible for a larger number of cells to deposit in the kidney scaffold, compared with the protocols used previously by us and others, but they also confirmed that the high perfusion pressure induces cell extravasation and accumulation in focal areas of the scaffold that are likely the result of dilated or disrupted tubular membranes.

Because results from cell seeding procedures show that overall the extent and location of recellularization is limited, one can argue that seeded cells can proliferate and regenerate the native kidney’s cellular organization. Next, we evaluated cell proliferation after seeding. Our results show that in the first two days after seeding, cells did proliferate, but this phenomenon slowed down significantly over time. There could be several explanations for the reduced cell proliferation over time, and these are beyond the aim of our study and worth investigating in future studies. One is that some seeded cells may start to differentiate, as we have previously shown^17^, thus reducing the proliferation rate. Another explanation may be related to non-optimal delivery of oxygen and cell nutrients. The overall result is that culturing seeded cells for a prolonged period of time as in our experimental conditions is unlikely to induce kidney cell numbers and the structure to recreate the native organ.

Recently, Ko and coworkers^18^ proposed another approach. These authors aimed to efficiently re-endothelialzed the vasculature of acellular porcine kidneys to completely cover the vascular ECM. They combined a static protocol with a ramping cell infusion protocol. They infused CD31 antibody to obtain conjugation with a decellularized vascular matrix before cell seeding. This strategy actually improved endothelial cell attachment and retention, leading to vascular patency of the whole kidney 4 hours after *in vivo* implantation. However, the brief implantation time does not allow us to conclude that blood coagulation is effectively prevented by this endothelialization strategy. Detailed evaluation of endothelial coverage, especially in the venous compartment, was not reported in this investigation.

Our present data clearly show that kidney recellularization with embryonic stem cells, and the infusion protocols we adopted, do not result in uniform and extensive cell deposition throughout the entire volume of the acellular scaffolds. We therefore considered which physical factors could potentially be responsible for difficulties with delivering cells uniformly along the acellular vasculature and behind the glomerular capillaries. We wondered whether the hydraulic permeability of the vessel walls of the decellularized scaffold might induce leakage of cell medium during cell seeding out of the vessel lumen, making it impossible to push cells further along the kidney vasculature. The results of our theoretical analysis show that the velocity of cell suspension is expected to slow down significantly during transit from arteries to arterioles and to the microcirculation. This is due to the combination of the natural branching of the vasculature and the leakage of medium out of the vessel wall. Thus, during the seeding procedure, cells in the glomerular capillaries must be almost immobile. Cell clusters formed in the glomerular capillaries due to the low speed cannot be pushed further down into peritubular capillaries or into the venous circulation. In addition, pressure cannot be increased through capillary occlusion, as fluid can easily escape through the arterial wall. These data suggest that there are physical barriers that impede the uniform seeding of cells into the scaffold’s circulation.

The experimental and theoretical results also indicate that increasing the flow rate in the renal artery during cell seeding perfusion (arriving to 20 ml/min of flow rate) does allow us to increase the delivery of cell suspension along the microcirculation but, instead of distributing uniformly into the venous side, cells were predominantly found in focal areas of the kidney parenchyma. The morphology of the structures occupied by the cells, which are importantly enlarged as compared to normal tubules, indicates that disruption of the tubular membrane may have occurred, likely for the high perfusion pressure. Another interpretation could be that, soon after seeding, mES cells manipulated the tubular basement membrane, but this is rather unlikely, given the short observation time.

Our investigation is limited to a single cell phenotype (mES cells) and to selected perfusion conditions in terms of cell suspension volume, and perfusion flow rate. Our results cannot rule out the possibility that more favorable results could be obtained by using other cell types (such as fetal or progenitor cells, as well as differentiated cells). Despite using different perfusion conditions, we cannot exclude the possibility that different experimental conditions could possibly allow more uniform and extensive seeding than we observed. Thus, different strategies to recellularize kidney scaffolds are worth investigating.

Based on these observations we can outline the most important hurdles to the very ambitious task of kidney regeneration. The first task must be to develop a technique for manipulating acellular kidney scaffolds to reduce or completely block hydraulic permeability of vascular and tubular membranes, to block fluid leakage, and to allow perfusion of cells in the entire kidney structure, especially in the venous circulation. Theoretically, this can be obtained using compounds that adhere to the ECM and increase its resistance to fluid filtration. In addition to this, cellularization of the vascular lumen with an endothelial cell layer, or with programmed stem cells, may also improve the results of cell seeding. This could eventually also favor seeding and the differentiation of cells delivered to the renal tubules.

Another important limiting factor for extensive recellularization of the acellular scaffolds is the number of cells that can be infused. Since there is evidence that the proliferation of seeded cells is rather limited, the number of cells that must be infused should equal that of the native kidneys. This means using 1 billion cells for the rat kidney, instead of the millions of cells used so far by us and others. In addition, our results clearly indicate that infusing such a massive number of cells will likely produce cell entrapment and consequently occlusion of the arterial and capillary circulation, rather than uniform cell distribution in the entire renal parenchyma. In the case of porcine kidney recellularization, this is an even more difficult problem, as the number of cells must be increased to hundreds of billions of cells. To obtain these huge numbers of cells in culture is very difficult with the available techniques, and new and more efficient methods for scaling up cell stem expansion and *in vitro* culture must be developed.

In conclusion, despite suggesting that *in vitro* recellularization of acellular kidney scaffold is feasible, our data clearly show that the extent and distribution of recellularization we obtained was rather limited. The hurdles, highlighted by our data are mainly the nonuniform and only focal cell seeding, and the limited cell proliferation with culture time. These observations are important because they can help to better shape expectations on the possibility to obtain kidney regeneration in the laboratory by scaffold recellularization.

## Methods

### Kidney recovery

The Institutional Animal Care and Use Committees of the Mario Negri Institute, Milan, Italy, approved all animal studies. Animal care and treatment were conducted in accordance with the institutional guidelines, in compliance with national (D.L.n.26, March 4, 2014), and international laws and policies (directive 2010/63/EU on the protection of animals used for scientific purposes). All rats were maintained in a pathogen–free facility with a 12-hour light/dark cycle and free access to standard diet and water. Healthy male Sprague-Dawley rats (Charles River S.p.A., Calco, Italy) weighing 250–400 g were used in this study. After anesthesia with isoflurane, a longitudinal abdominal incision was made and the left kidney, aorta, vena cava and ureter were identified. The left kidney was freed from surrounding fat and mobilized. Firstly, the ureter was cannulated with a PE10 catheter (Becton, Dickinson and Company, Franklin Lakes, New Jersey) and the renal artery with a PE50 catheter (Becton, Dickinson and Company). Finally the renal vein was cannulated with a 14-gauge cannula (Becton, Dickinson and Company). The kidney was then arterially perfused using saline solution (NaCl 0.9%, SALF SpA, Bergamo, Italy) containing a vasodilator (nitroprusside, 10^−4^ M, Sigma-Aldrich Co. LLC, St Louis, MO) to remove blood. After blanching, the kidney was harvested and kept immersed in saline solution.

### Rat kidney decellularization

Each kidney was initially perfused with a solution of 1% sodium dodecyl sulfate (SDS, Sigma-Aldrich Co. LLC) in distilled water through the arterial cannula that was connected to a peristaltic pump. SDS was perfused for 6 hrs at a flow rate of 0.4 ml/min. Then, decellularized scaffolds were gently washed with distilled water and perfused by phosphate buffer saline (PBS, Life Technologies Italia, Monza, Italy) containing 1% penicillin/streptomycin (Life Technologies Italia) to remove all cellular debris and chemical residues. Hydraulic pressure was continuously recorded by a pressure transducer (WPI Inc., Sarasota, FL), a digital data acquisition system MP150 and AcqKnowledge software (BIOPAC Systems, Inc., Goleta, CA). Physiological pressure (assumed to be in the range 110–115 mmHg for the rat)^24^ was maintained for the entire duration of the decellularization process. Before cell seeding, the scaffold was perfused with the same medium used for cell culture devoid of LIF at 37 °C for 2 hours to provide nutrients and establish the right pH in the scaffold.

### Orthotopic transplantation of acellular scaffold

Renal acellular scaffolds were orthotopically transplanted using a well-established surgical procedure^41^. N = 5 recipient Lewis rats (Charles River S.p.A., Calco, Italy) were anesthetized with a mixture of 5% Isoflurane and 2 l/min oxygen for induction and 1.5% Isoflurane and 2 l/min oxygen for maintenance. The animals were then prepared by removal of the left kidney. An anastomosis was created between the recipient and donor renal artery as well as the renal vein with end-to-end anastomosis. Vascular clamps were released after 30 min. Reconstruction of the ureter was performed with a stent. At the end of the transplant 0.05 mg/kg of the analgesic Buprenorphine were administered subcutaneously. After 1 hour heparin was intraperitoneally administered to the animal (25 UI). For 7 days after the procedure Acetylsalicylic acid (1 mg/kg) was administered per os. On postoperative days 3, 7 and 21, the animals were sacrificed using a lethal anesthetic drug injection and the grafts removed for histological analysis.

### Murine ES cell culture

The R1 mouse pluripotent embryonic stem (mES) cells were obtained following MTA agreement from A. Nagy laboratory^42^ (Mount Sinai Institute, Toronto, Canada). mES cells are defined a cell line because they can propagate indefinitely in culture without being conventionally immortalized. R1 mES cells were cultured and passaged every two days on mitotically inactivated mouse embryonic fibroblasts (MEFs; E13.5; strain 129) in proliferation medium DMEM supplemented with 1 mM sodium pyruvate, 100 μM non-essential amino acids, 2 mM L-glutamine, 100 U/ml penicillin–100 μg/ml streptomycin, 0.1 mM β-mercaptoethanol, 10% ES cell qualified FBS (all products were obtained from Life Technologies Italia, Italia) and 1000 U/ml leukemia inhibitory factor (LIF ESGRO, Merck Millipore, Billerica, MA).

### Cell seeding with mES cells

For cell seeding, perfusion was halted, and three suspensions of culture medium (3 ml) each containing 5 × 10^6^ mES cells (15 × 10^6^ cells in total) were infused antegrade through the arterial cannula connected to a syringe pump (Harvard Apparatus, Holliston, MA) at 1 ml/min of flow rate. Each infusion was followed by recirculation of culture medium for 10 minutes at 0.4 ml/min.

In a second series of experiments, to infuse cells into the tubular space, following the procedure above, we infused mES cells via the ureter too. Prior to ureteral seeding, vacuum was applied to the chamber, lowering the gauge pressure to −50 mmHg. Three cell suspensions (containing 5 × 10^6^ mES cells each, 15 × 10^6^ cells in total) were infused through the ureteral cannula at 1 ml/min. After injection, a recirculation of culture medium for 10 minutes at 0.4 ml/min was also applied under negative pressure.

To further improve seeding efficiency, another infusion protocol was adopted. The scaffolds were seeded with a triple cell infusion through the renal artery, ureter and renal vein, respectively. First, 15 × 10^6^ mES cells were injected through the renal artery, as described above. The second step was the perfusion of 15 × 10^6^ mES cells via the ureter, applying a negative pressure outside the kidney. Last, three cell suspensions (containing 5 × 10^6^ mES cells each, 15 × 10^6^ cells in total) were seeded through the venous cannula following the same procedure of the arterial infusion. This procedure enabled the repopulation of the kidney scaffolds with 45 × 10^6^ mES cells in total.

Finally, because in a recent report^9^ rather efficient cell seeding was obtained adopting high pressure during cell infusion, we also adopted a similar protocol. mES cells (40 × 10^6^) were suspended in 2 ml and injected through a syringe pump through the renal artery at a flow rate of 1 ml/min. The scaffold was then immediately perfused at a flow rate of 21 ml/min for 15 min to push cells further into the scaffold. The flow rate was then decreased to 4 ml/min and maintained in perfusion culture.

### Culture of seeded mES cells in kidney scaffolds

After cell seeding, kidney scaffolds were kept in a cell incubator and continuously perfused with recirculating culture medium through the renal artery for 24 and 72 hours at a flow rate of 0.4 ml/min. Culture medium was allowed to equilibrate with 5% CO_2_ and 95% air by flowing through silicone tubing before reaching the cannulated renal artery. The ureter and vein were allowed to drain passively during the perfusion culture. For the infusion protocol at elevated pressure the medium flow rate through the renal artery was maintained at 4 ml/min for 24 hours, 72 hours and 7 days.

### Histological analysis

At the end of perfusion, recellularized kidneys were cut into four transversal sections. Two tissue samples were maintained in Duboscq-Brazil fixative (Diapath S.p.A., Bergamo, Italy) overnight for histological analysis and two were fixed in periodate (Merck KGaA, Darmstadt, Germany) -lysine (Sigma-Aldrich Co. LLC) -paraformaldehyde (Electron Microscopy Science, Hatfield, PA) fixative (PLP) at 4 °C overnight for immunofluorescence analysis. For histological analysis the sections were dehydrated in ascending concentrations of alcohol and embedded in paraffin. For each paraffin block of tissue, 3 μm sections were obtained and stained with hematoxylin and eosin (Bio Optica Milano S.p.A., Milano, Italy). Tissue sections of hematoxylin and eosin staining were examined on Axiovision light microscope (Zeiss, Jena, Germany). For morphometrical quantification in average 9 sections (from 6 to 12) in three to five kidney samples for each seeding condition were analyzed. For each sampling section, 20 fields were sequentially digitized using an x40 objective. The volume density of seeded cells was estimated by point counting using an orthogonal grid with 29 × 22 lines digitally overlaid on the stained section image. The number of grid points hitting the cells and the number of grid points hitting the empty scaffold were counted. Volume density was calculated as the percent ratio between grid points in the positive areas over >25,000 of total points in the field. With this sample size the expected relative probable error is in the range of 3–4% of the mean volume density.

### Cell proliferation

To determine the proliferation of injected cells within the kidney scaffolds, immunofluorescence staining was performed using Ki-67 antigen staining in n = 3 kidneys for cell seeding condition. Briefly, after being kept at 4 °C overnight, tissue samples fixed in PLP were immersed in 30% sucrose overnight, frozen in liquid nitrogen and cut into 3 μm sections. Frozen sections were then incubated with mouse monoclonal Ki-67 (Novocastra Laboratories Ltd, Newcastle, UK, diluted 1:100), followed by donkey anti-rabbit IgG Cy3 conjugate (Li StarFish S.r.l., Milano, Italy, diluted 1:50). Staining with DAPI 1 mg/mL (Sigma-Aldrich Co. LLC) and Fluorescein Wheat Germ Agglutinin (Vector Laboratories, Inc., Burlingame, CA, diluted 1:400) for 20 min was performed for cell nuclear staining and matrix morphology assessment, respectively. Fluorescence images were acquired by Apotome Axio Imager Z2 (Carl Zeiss Inc., Göttingen, Germany). The percentage of KI67 positive cells per field over total DAPI positive nuclei was counted in 50 non-overlapping random images per kidney section obtained at 24 and 72 hours of perfusion.

### Quantification of cell suspension delivery

In an attempt to document the efficiency of cell delivery into the acellular scaffolds, and the fate of the cell suspension in the scaffold volume, we performed a series of additional experiments to infuse a controlled volume of complete cell culture medium without LIF into the renal artery of acellular kidneys and collected the effluent from the renal vein. The cell medium was isotonic, with pH 7.2, osmolarity of 276 mosm/kg and cinematic viscosity of 0.89 cP. Due to the hydraulic permeability of the extracellular matrix, we collected the fluid filtered outside the circulation and permeating into the tubules and then into the ureter as well as fluid permeating out of the ECM scaffolds. Different flow rates were used in these perfusion experiments from 0.4 to 1.0 ml/min.

### Theoretical simulation of kidney scaffold perfusion

With the aim of estimating the effective distribution of the perfusion fluid in the scaffold circulation, and into the microvessels, we adopted a theoretical model to simulate flow velocities in the blood vessels and throughout the permeable matrix of the vessel wall. For this simulation we used the data that we obtained on flow rate entering into the renal artery and exiting the scaffolds, as measured during a series of perfusion experiments we performed at different infusion rates (1, 10 and 20 ml/min, respectively), recording a hydraulic pressure drop. A structural model of the kidney vasculature was then derived from the work of Nordsletten *et al*.^43^, who reported detailed data on the dimensions (mean diameter and length) of arterial and venous segments of the entire kidney circulation, derived from a microCT investigation using a rat kidney. On the basis of this structural model, and considering the flow of cell medium laminar in arterial and venous segments, we developed a theoretical model that allows us to compute flow velocity in all individual vascular segments, assuming an input value of the hydraulic permeability of the extracellular matrix membrane of kidney vessels. The basic equations of the theoretical model, and its experimental validation, are reported in the Appendix (Supplementary Material).

### Statistical Analysis

Data are expressed as mean ± standard deviation (SD). Statistical analysis was performed using analysis of variance (Prism 6.0; GraphPad Software Inc, San Diego, CA). Bonferroni post-hoc analysis was adopted to estimate statistical significance between group comparisons. Statistical significance was defined as p < 0.05.

## Additional Information

**How to cite this article:** Remuzzi, A. *et al*. Experimental Evaluation of Kidney Regeneration by Organ Scaffold Recellularization. *Sci. Rep.* **7**, 43502; doi: 10.1038/srep43502 (2017).

**Publisher's note:** Springer Nature remains neutral with regard to jurisdictional claims in published maps and institutional affiliations.

## Supplementary Material

Supplementary Material

Supplementary Video

## Figures and Tables

**Table 1:** Percentage of proliferating Ki-67 positive cells in recellularized kidneys.

	24 h	72 h	7 days
RA + RV + U	57.5 ± 3.1%	37.7 ± 4.9%^a^	—
HP	50.9 ± 11.2%	51.8 ± 2.1%	23.7 ± 13.1%^b,c^

Data are mean ± SD. Abbreviations: RA, Cell infusion into the renal artery; RV, Cell infusion into the renal vein; U, Cell infusion into the ureter: HP, Cell infusion into the renal artery at high pressure.

^a^p < 0.01 vs. 24 h, ^b^p < 0.05 vs. 24 h, ^c^p < 0.01 vs. 72 h.

## References

[b1] Thomas B. . Maintenance Dialysis throughout the World in Years 1990 and 2010. J Am Soc Nephrol 26, 2621–2633 (2015). 26209712 10.1681/ASN.2014101017 PMC4625679

[b2] Liyanage T. . Worldwide access to treatment for end-stage kidney disease: a systematic review. Lancet 385, 1975–1982 (2015). 25777665 10.1016/S0140-6736(14)61601-9

[b3] Couser W. G., Remuzzi G., Mendis S. & Tonelli M. The contribution of chronic kidney disease to the global burden of major noncommunicable diseases. Kidney Int 80, 1258–1270 (2011). 21993585 10.1038/ki.2011.368

[b4] Winkelmayer W. C., Weinstein M. C., Mittleman M. A., Glynn R. J. & Pliskin J. S. Health economic evaluations: the special case of end-stage renal disease treatment. Med Decis Making 22, 417–430 (2002). 12365484 10.1177/027298902236927

[b5] Hart A. . Kidney. Am J Transplant 16 Suppl 2, 11–46 (2016). 10.1111/ajt.13666 PMC5541687 26755262

[b6] Uzarski J. S., Xia Y., Belmonte J. C. & Wertheim J. A. New strategies in kidney regeneration and tissue engineering. Curr Opin Nephrol Hypertens 23, 399–405 (2014). 24848937 10.1097/01.mnh.0000447019.66970.ea

[b7] Soto-Gutierrez A., Wertheim J. A., Ott H. C. & Gilbert T. W. Perspectives on whole-organ assembly: moving toward transplantation on demand. J Clin Invest 122, 3817–3823 (2012). 23114604 10.1172/JCI61974 PMC3484436

[b8] Ross E. A. . Embryonic stem cells proliferate and differentiate when seeded into kidney scaffolds. J Am Soc Nephrol 20, 2338–2347 (2009). 19729441 10.1681/ASN.2008111196 PMC2799178

[b9] Caralt M. . Optimization and critical evaluation of decellularization strategies to develop renal extracellular matrix scaffolds as biological templates for organ engineering and transplantation. Am J Transplant 15, 64–75 (2015). 25403742 10.1111/ajt.12999 PMC4276475

[b10] Karczewski M. & Malkiewicz T. Scaffolds from surgically removed kidneys as a potential source of organ transplantation. Biomed Res Int 2015, 325029 (2015). 25756044 10.1155/2015/325029 PMC4338377

[b11] Scarrit M. E., Pashos N. C. & Bunnel B. A. A review of cellularization strategies for tissue engineering of whole organs. Front Bioeng Biotechnol 3, 43 (2015). 25870857 10.3389/fbioe.2015.00043 PMC4378188

[b12] Fountain H. Rat Kidneys made in Lab Point to Aid for Humans. In The New York Times (2013).

[b13] Kridel R. W., Foda H. & Lunde K. C. Septal perforation repair with acellular human dermal allograft. Arch Otolaryngol Head Neck Surg 124, 73–78 (1998). 9440784 10.1001/archotol.124.1.73

[b14] Cho S. W. . Vascular patches tissue-engineered with autologous bone marrow-derived cells and decellularized tissue matrices. Biomaterials 26, 1915–1924 (2005). 15576165 10.1016/j.biomaterials.2004.06.018

[b15] Burch P. T. . Clinical performance of decellularized cryopreserved valved allografts compared with standard allografts in the right ventricular outflow tract. Ann Thorac Surg 90, 1301–1305 discussion 1306 (2010). 20868833 10.1016/j.athoracsur.2010.05.024

[b16] Song J. J. . Regeneration and experimental orthotopic transplantation of a bioengineered kidney. Nat Med 19, 646–651 (2013). 23584091 10.1038/nm.3154 PMC3650107

[b17] Bonandrini B. . Recellularization of well-preserved acellular kidney scaffold using embryonic stem cells. Tissue Eng Part A 20, 1486–1498 (2014). 24320825 10.1089/ten.tea.2013.0269 PMC4011423

[b18] Ko I. K. . Enhanced re-endothelialization of acellular kidney scaffolds for whole organ engineering via antibody conjugation of vasculatures. Technology 2, 1–9 (2014).

[b19] Peloso A. . Current achievements and future perspectives in whole-organ bioengineering. Stem Cell Res Ther 6, 107 (2015). 26028404 10.1186/s13287-015-0089-y PMC4450459

[b20] Petrosyan A. . Decellularized Renal Matrix and Regenerative Medicine of the Kidney: A Different Point of View. Tissue Eng Part B Rev (2016). 10.1089/ten.TEB.2015.0368 26653996

[b21] Katari R. . Renal bioengineering with scaffolds generated from human kidneys. Nephron Exp Nephrol 126, 119 (2014). 24854653 10.1159/000360684

[b22] Lin Y. Q. . Kidney bioengineering in regenerative medicine: An emerging therapy for kidney disease. Cytotherapy 18, 186–197 (2016). 26596504 10.1016/j.jcyt.2015.10.004

[b23] Moon K. H., Ko I. K., Yoo J. J. & Atala A. Kidney Diseases and Tissue Engineering. Methods (2015). 10.1016/j.ymeth.2015.06.020 26134528

[b24] Osborn J. W. & Fink G. D. Region-specific changes in sympathetic nerve activity in angiotensin II-salt hypertension in the rat. Exp Physiol 95, 61–68 (2010). 19717492 10.1113/expphysiol.2008.046326 PMC2856071

[b25] Peloso A. . Renal Extracellular Matrix Scaffolds From Discarded Kidneys Maintain Glomerular Morphometry and Vascular Resilience and Retains Critical Growth Factors. Transplantation 99, 1807–1816 (2015). 26018349 10.1097/TP.0000000000000811

[b26] Gorji M., Alipanah M., Shateri M. & Farnad E. Analytical solution for laminar flow through leaky tube. Appl. Math. Mech. -Engl. Ed. 32, 69–74 (2011).

[b27] Sadri A. R., Jeschke M. G. & Amini-Nik S. Advances in Liver Regeneration: Revisiting Hepatic Stem/Progenitor Cells and Their Origin. Stem Cells Int 2016, 7920897 (2016). 26798363 10.1155/2016/7920897 PMC4699025

[b28] Mae S. & Osafune K. Kidney regeneration from human induced pluripotent stem cells. Curr Opin Organ Transplant 20, 171–177 (2015). 25856179 10.1097/MOT.0000000000000170

[b29] Katari R. S., Peloso A. & Orlando G. Tissue engineering. Adv Surg 48, 137–154 (2014). 25293612 10.1016/j.yasu.2014.05.007

[b30] Takahashi K. & Yamanaka S. Induction of pluripotent stem cells from mouse embryonic and adult fibroblast cultures by defined factors. Cell 126, 663–676 (2006). 16904174 10.1016/j.cell.2006.07.024

[b31] Gilbert T. W., Sellaro T. L. & Badylak S. F. Decellularization of tissues and organs. Biomaterials 27, 3675–3683 (2006). 16519932 10.1016/j.biomaterials.2006.02.014

[b32] Baptista P. M. . Whole organ decellularization - a tool for bioscaffold fabrication and organ bioengineering. Conf Proc IEEE Eng Med Biol Soc 2009, 6526–6529 (2009). 19964173 10.1109/IEMBS.2009.5333145

[b33] Badylak S. F., Weiss D. J., Caplan A. & Macchiarini P. Engineered whole organs and complex tissues. Lancet 379, 943–952 (2012). 22405797 10.1016/S0140-6736(12)60073-7

[b34] Crapo P. M., Gilbert T. W. & Badylak S. F. An overview of tissue and whole organ decellularization processes. Biomaterials 32, 3233–3243 (2011). 21296410 10.1016/j.biomaterials.2011.01.057 PMC3084613

[b35] Guan Y. . The effective bioengineering method of implantation decellularized renal extracellular matrix scaffolds. Oncotarget 6, 36126–36138 (2015). 26418881 10.18632/oncotarget.5304 PMC4742166

[b36] Peloso A. . Creation and implantation of acellular rat renal ECM-based scaffolds. Organogenesis 11, 58–74 (2015). 26186418 10.1080/15476278.2015.1072661 PMC4594518

[b37] Zambon J. P. . Kidney regeneration: Where we are and future perspectives. World J Nephrol 3, 24–30 (2014). 25332894 10.5527/wjn.v3.i3.24 PMC4202490

[b38] Salvatori M., Peloso A., Katari R. & Orlando G. Regeneration and bioengineering of the kidney: current status and future challenges. Curr Urol Rep 15, 379 (2014). 24375058 10.1007/s11934-013-0379-9

[b39] Petrosyan A. . Understanding the bioactivity of stem cells seeded on extracellular matrix scaffolds produced from discarded human kidneys: a critical step towards a new generation bio-artificial kidney. CellR4 3, 1–15 (2005).

[b40] Katari R. . Tissue-Engineering Approaches to Restore Kidney Function. Curr Diab Rep 15, 69 (2015). 26275443 10.1007/s11892-015-0643-0

[b41] Benigni A. . Adeno-associated virus-mediated CTLA4Ig gene transfer protects MHC-mismatched renal allografts from chronic rejection. J Am Soc Nephrol 17, 1665–1672 (2006). 16641148 10.1681/ASN.2006010090

[b42] Nagy A., Rossant J., Nagy R., Abramow-Newerly W. & Roder J. C. Derivation of completely cell culture-derived mice from early-passage embryonic stem cells. Proc Natl Acad Sci USA 90, 8424–8428 (1993). 8378314 10.1073/pnas.90.18.8424 PMC47369

[b43] Nordsletten D. A., Blackett S., Bentley M. D., Ritman E. L. & Smith N. P. Structural morphology of renal vasculature. Am J Physiol Heart Circ Physiol 291, H296–309 (2006). 16399870 10.1152/ajpheart.00814.2005

