# Supplementary material for: Experimental Evaluation of Kidney Regeneration by Organ Scaffold Recellularization
Source: Sci Rep. 2017 Mar 7;7:43502. doi: 10.1038/srep43502 (PMC5339865; doi:10.1038/srep43502)
Supplement: Supplementary Material [file srep43502-s1.docx]

**Appendix & Additional Material**

*Manuscript*:

Experimental Evaluation of Kidney Regeneration by Organ Scaffold Recellularization

Andrea Remuzzi, Marina Figliuzzi, Barbara Bonandrini, Sara Silvani, Nadia Azzollini, Roberta Nossa, Ariela Benigni and Giuseppe Remuzzi

**APPENDIX**

In order to estimate the distribution of perfusion fluid within the scaffold during the cell seeding procedure, we developed a mathematical model based on the geometrical parameters of kidney vasculature reported in literature by Nordsletten and co-authors^1^. These Authors used detailed microCT acquisition and 3D reconstruction of the kidney vascular (using a contrast media) then they derived by digital image processing the topology and dimensions of the arterial and venous vascular segments. According to the data set they provided, the rat kidney vascular network is divided in different vessel orders (hierarchical levels) for the arterial and venous tree. For each level, the number of elements, the mean radius and mean length have been estimated. To obtain a complete geometrical description of the vascular network, from the renal artery to the renal vein, we assumed the number, length and radius of glomerular capillary segments that have not been identified by Nordsletten et al. for not enough resolution of the microCT images^1^. We estimated these later values according to the 3D reconstruction of the rat glomerular capillary we reported previously^2^. The final data set of the entire kidney vasculature we assumed is reported in **Table A1.**

Using these geometrical parameters we calculated the pressure drop for a given the flow rate in each vessel segment order, using Poiseuille equation

$P_{out,i}= P_{in,i}- \frac{8\mu\Delta x_{i}}{\pi R_{i}^{4}} \cdot Q_{i}$ (1)

where, for each hierarchical level *i*, *P_out,i_*  is the output pressure, *P_in,i_* the inlet pressure, *Q_i_* the flow rate, *μ* the fluid viscosity, Δ*x_i_* the average vessel length and *R_i_* the mean vessel radius. Initially, we also assumed that the vessel wall of the arterial and venous segments is not permeable, i.e. no filtration across vessel wall. The flow rate at the entrance of the vessel network was assumed equal to the perfusion flow rate, then for each hierarchical level we calculated the flow rate in each segment dividing the inlet flow by the number of vessel segments of the level considered. This calculation was repeated up to the glomerular capillary. In the venous tree, the flow rate was calculated similarly, increasing the flow of individual vessel segment order according to the assumed number. For the numerical procedure, we initially adopted an input value of the perfusion pressure into the renal artery (*P_in_*) and calculated the outflow pressure in the renal vein (*P_out_*) for the assumed flow rate (from experimental condition). We then iterated this calculation until the outflow pressure was equal zero.

To validate the calculation of the pressure drop of the entire vasculature in case of impermeable wall, and the relative flow distribution along the network, we perfused native kidneys (n=3) harvested from rats of comparable age and weight, as those used for kidney scaffold production. As shown in **Figure A1** the total pressure drop measured by us and that reported in literature for a similar investigation, are comparable to that calculated by the present network model for different flow rates perfused in the renal artery of native rat kidneys.

On the basis of this agreement, we extended the theoretical model to simulate also the perfusion of permeable basement membranes with consequent fluid filtration across the vessel wall. To this purpose we assumed that the flow rate inside a vessel of a given hierarchical level is not constant along the vessel axis, but it decreases gradually due to filtration, implementing in our network model the analytical solution described by Gorji and co-authors^3^ for permeable fluid flow in a permeable tube. We assumed that the flow is laminar along all blood vessels of the scaffold and that the geometry of each hierarchical level is equivalent to that of a straight tube with uniform radius, respectively the average length (*L*) and radius (*R*). Assuming *z* as the position along the tube axis, the velocity (*V*) and the pressure depend from local position

$V=[V_{r}\left( r,z \right), V_{z}\left( r,z \right)]$ (2)


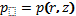
 $p=p\left( r,z \right)$ (3)

We then assumed that the flow field is described by the simplified Navier-Stokes equation and by the continuity equation

$-\frac{\partial p}{\partial z}+ \mu\left( \frac{1}{r}\frac{\partial}{\partial r}\left( r\frac{\partial V_{r}}{\partial r} \right)+ \frac{\partial^{2}V_{z}}{\partial^{2}r} \right)=0$ (4)

$\frac{1}{r} \frac{\partial}{\partial r} \left( r V_{r} \right)+ \frac{\partial V_{z}}{\partial z}=0$ (5)

where
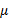

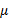
 is the fluid dynamic viscosity. The flux of fluid filtered across the vessel wall depends from the transmural pressure gradient and the hydraulic permeability of the tube wall, and decreases along the tube axis (*z*) for the expected change in pressure drop

$j\left( z \right)= k\left( p\left( z \right)-p_{0} \right)$ (6)

where
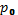

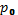
 is the pressure outside the tube and
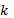

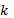
 is the hydraulic permeability coefficient that is the product between the Darcy permeability
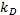

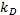
 and the wall tube surface area
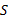

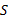
. Using the analytical solution proposed by Gorji et al. ^3^, the amount of fluid filtered by each vessel in a given hierarchical level is given by

$Q_{f}=2\pi Rk \left\{ P_{in}L- \frac{\mu U_{0}}{2\beta^{2}} \left[ exp\left( -4\beta\frac{L}{R} \right)-1 \right]-2\frac{\mu U_{0}}{2R}L \right\}$ (7)

where

$\beta= \left( \frac{k\mu}{R} \right)^{0.5}$ (8)

and the flow rate and mean velocity, at the end-side of the tube of level *i*, are given by


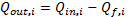
 $Q_{out,i}= Q_{in,i}-Q_{f,i}$ (9)

and


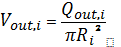
 $V_{out,i}= \frac{Q_{out,i}}{\pi R_{i}^{2}}$ (10)

respectively. Since the permeability coefficient is unknown, we used an iterative procedure starting from an input value of *k* and then we changed this value until the calculated total filtered flow rate was equal to the average values determined experimentally.

**REFERENCE**

1. Nordsletten, D.A., Blackett, S., Bentley, M.D., Ritman, E.L. & Smith, N.P. Structural morphology of renal vasculature. Am J Physiol Heart Circ Physiol 291, H296-309 (2006).

2. Antiga, L., Ene-Iordache, B., Remuzzi, G. & Remuzzi, A. Automatic generation of glomerular capillary topological organization. Microvasc Res 62, 346-354 (2001).

3. Gorji, M., Alipanah, M., Shateri, M. & Farnad, E. Analytical solution for laminar flow through leaky tube. Appl. Math. Mech. -Engl. Ed. 32, 69-74 (2011).

**Table A1**. Geometrical parameters of rat kidney vasculature.

| **Vessel** | **Hierarchical**  **level** | **Mean radius**  (*µm*) | **Mean length**  (*µm*) | **Number of**  **elements** |
| --- | --- | --- | --- | --- |
|  | 0 | 216.1 | 3,000 | 1 |
|  | 1 | 191.4 | 11,440 | 3 |
|  | 2 | 139.8 | 8,975 | 6 |
|  | 3 | 86.1 | 2,516 | 24 |
|  | 4 | 53.9 | 1,031 | 90 |
| Arteries | 5 | 44.2 | 511 | 247 |
|  | 6 | 39.3 | 1,001 | 578 |
|  | 7 | 29.9 | 656 | 1,245 |
|  | 8 | 20.1 | 404 | 4,373 |
|  | 9 | 13.9 | 423 | 13,070 |
| Afferent arterioles* | 10 | 10.1 | 312 | 29,566 |
| Glomerular Capillary Segments* | 11 | 5.0 | 150 | 591,320 |
| Efferent arterioles* | 12 | 6.1 | 300 | 29,566 |
|  | 11 | 10.8 | 155 | 68,564 |
|  | 10 | 14.7 | 248 | 30,659 |
|  | 9 | 26.2 | 315 | 9,258 |
|  | 8 | 40.1 | 625 | 2,926 |
|  | 7 | 50.3 | 820 | 1,210 |
| Veins | 6 | 69.2 | 1,054 | 418 |
|  | 5 | 114.1 | 1,147 | 139 |
|  | 4 | 177.0 | 1,695 | 38 |
|  | 3 | 285.6 | 6,131 | 9 |
|  | 2 | 428.0 | 3,091 | 4 |
|  | 1 | 603.8 | 3,123 | 2 |
|  | 0 | 850.0 | 6,000 | 1 |

All values for vessel radius length and number for each level are adapted form Nordsletten et al. (1). * Values adapted from Antiga et al. (2).


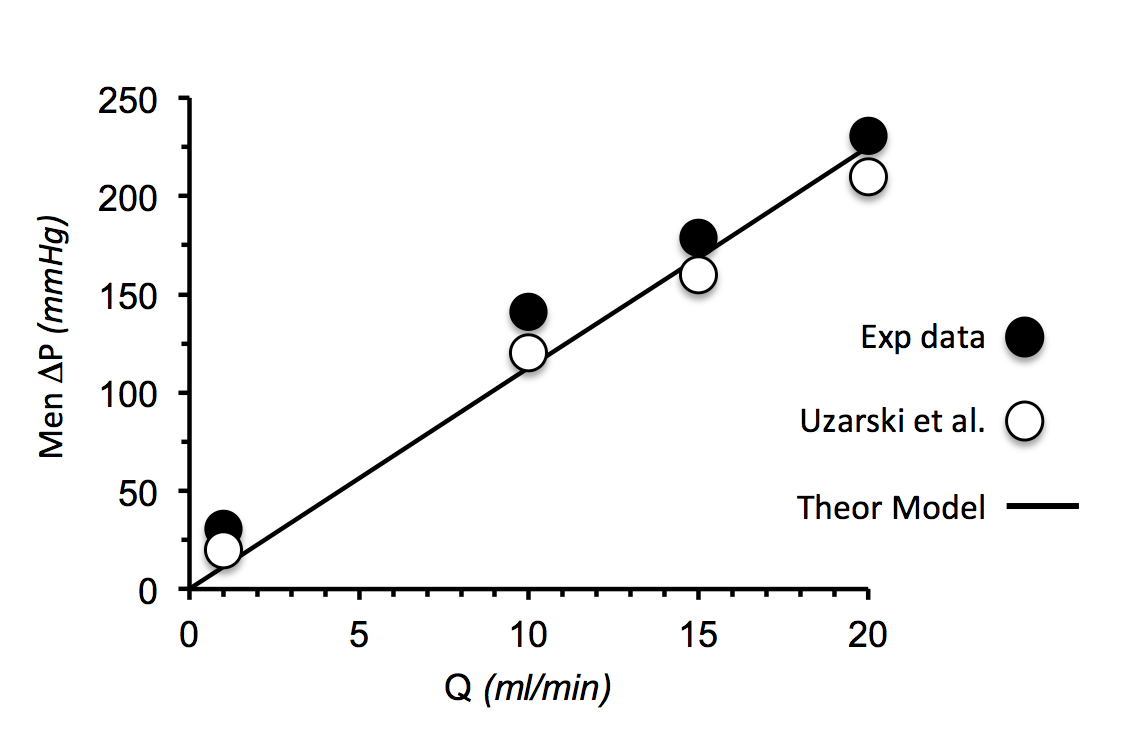


**Figure A1** - Total pressure drop of native rat kidneys at different perfusion rates (Q). Measured pressure drop ( ); data reported in literature for native rat kidney ( o ) and values calculated with the theoretical model (^_____^).

**Additional Material**

To better understand the localization of the cells infused through the renal vein, 60 serial sections of the cortical area of recellularized kidney (3 μm thickness) were obtained and stained with hematoxylin and eosin. The middle section of the series was labeled as “starting section”. In the starting section tubular capillaries were randomly identified and images were digitally acquired at light microscopy, above and below the starting section. Acquired images were registered and resized using plugins of ImageJ software (NIH, USA - [imagej.nih.gov](https://imagej.nih.gov)) and the image stack saved in .vtk format. For 3D visualization of the cortical volume, we processed the image stack using the software ParaView (KitWare Inc. New York USA, www.paraview.org) and generated a 3D view, and its rotation. Low intensity values of tubular lumen were assumed to be transparent for better identification of the spatial relation between seeded cells and tubular membranes.


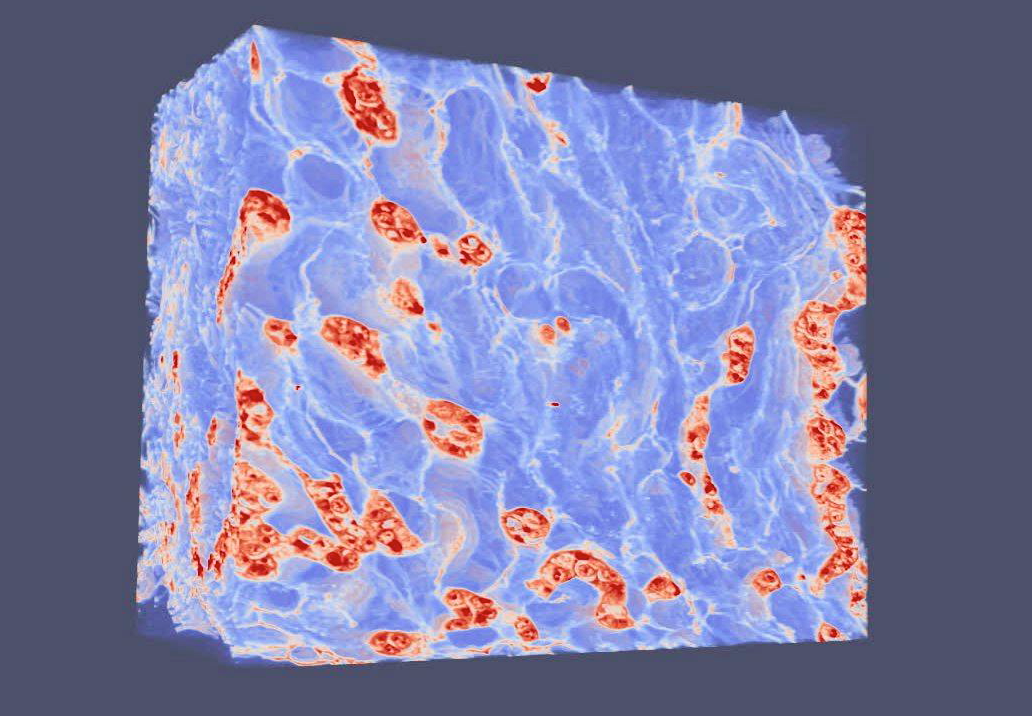


**Figure A2 –** (link to the movie.avi)
